# The Therapeutics of Headache

**Published:** 1877-02-20

**Authors:** A. A. Smith


### THE THERAPEUTICS OF HEADACHE.

A Lecture Delivered at Bellevue Hospital Med. College.
By A. A. SMITH, M.D.

Gentlemen—We take up to-day the therapeutics of certain forms of headache, a very important subject. Headache may be divided into organic and functional; but I believe you will get a better idea of the treatment by dividing the cases according to the causes.

You will remember we took up purely neuralgic headache at the last lecture.

A headache, when due to nervous disturbance, such as occurs in hysterical or excitable subjects, if associated with plethora, often yields to a saline cathartic. The most agreeable is the solution of citrate of magnesia, and should be given, a full bottle of it on an empty stomach. In addition, it is well to give one of the bromides combined with valerian. The following prescription I frequently use:

R. Sodii bromid..........dr.vi.
Elix. valer. amm.....oz.iv.

M. Sig. dr.i. every hour until relieved.

If such nervous headache be associated with anæmia, after relieving the immediate attack with the bromide and valerian prescription, give iron, and give it for weeks, until there is a decided improvement in the patient's condition. Always give the iron after meals. In these anæmic cases it is often advisable to stimulate the heart's action. For this purpose I have found the following useful;

R. Amm. muriat............oz.ss.
Tinct. actaæ racemos.,
Aquæ.. ....... ......aa..oz.iij.

M. Sig. dr.ij. after meals in a wineglass of water.

If there be despondency and depression of spirits, phosphorus, with nux vomica, is a good combination. The unpleasant taste of the phosphorus has been overcome by being made into sugar-coated or gelatine-coated pills. I frequently prescribe a pill containing phosphorous gr. 1/[illegible], with ext. nux vomica, gr. $\frac{1}{8}$ t. i. d., with the happiest results. The pills can be obtained of any reliable druggist This despondency is apt to occur in those who have been overworked mentally, or are harrassed by business cares, or who suffer great mental anxiety. If, in addition to these symp toms there be sleeplessness, I employ the following pill:

R. Camph. pulv.......... gr.xxv.
Ext. cannab. ind......gr.x.
Ext. hyoscyami.......gr.xx.

M. Div. in pill No. x.

Tig. One at night. Repeat in two hours if necessary to produce sleep.

It is important to attend to the general health of the patient. Remove all causes of excitement; encourage exercise in the open air; let the food be simple but nutritious; let the sleeping-room be large and well ventilated; in short, let the patient be surrounded by the best possible hygienic influences. These general remarks will apply to almost all forms of headache.

### SICK-HEADACHE.

I usually recognize two forms of sick-headache (so-called), the one neuralgic in character, as hemicrania and trifacial neuralgia, the other a dyspeptic headache. In the neuralgic variety the pain in the head precedes the nausea, while in the dyspeptic variety the pain in the head succeeds the dyspeptic symptoms. In the neuralgic, vomiting does not relieve the pain, while in the dyspeptic an emetic or laxative often removes the pain in the head by removing the cause. In addition to the treatment given in a previous lecture for neuralgic headache, which often occurs at intervals of a few days, or a week or two, sometimes coming on at sunrise and disappearing at sunset, I have good results from the use of guarana, or paullinia sorbillis, as it is sometimes called. I give it usually in powder, grains fifteen every fifteen minutes, until six doses have been taken. It is best given in a little sweetened water; and if six doses do not relieve, do not continue it; it will probably not relieve. It is well to give these powders in any headache (not malarial) of long standing and prone to return at certain intervals.

MALARIAL HEADACHE.

Malarial poison may prodnce pain in any portion of the head, but the most frequent locations are the sub-occipital region, the frontal, and on either side (hemicrania). Begin your treatment by the use of quinine. If distinctly periodical, give ten or fifteen grains two or three hours before the expected attack. It may be necessary to push the quinine in divided doses until cinchonism is produced, and kept up for several days, and then gradually diminish the dose. If the pain still continues to recur, and it frequently will, resort to arsenic and belladonna, five-drop doses each of Fowler's solution and tincture belladonna, after meals, increasing the Fowler's one drop each day until œdema arsenicalis is produced. This will seldom fail to give relief.

HEADACHE FROM GOUT.

I have found the following prescription beneficial in a headache dependent on gout:

R. Vin. colch. sem............dr.iij.
Lithii bromide.. ...........oz.ss.
Syr. zingib...............oz.ss.
Aq. cinnamonii, q. s. ad..oz.vi.

M. Sig. oz.ss. in a tumbler of Vichy water every four hours.

Such patients will be benefited by the regulation of the hygiene, tonics, a partial discontinuance of stimulants, particularly those which have been found by experience to aggravate the gouty symptoms.

SYPHILITIC HEADACHE.

It is hardly necessary that I should tell you that the headache of syphilis is more severe at night, and is quite apt to awaken the patient after twelve, by its increasing severity. The use of calomel in one-tenth grain doses every hour, for twelve hours immediately preceding the time that it awakens the patient, gives more rapid relief than the ordinary constitutional treatment. The calomel treatment may be continued for two or three days, and then stopped, and iodide of potassium given. I usually begin the iodide in fifteen-grain doses after meals, and gradually increase it until iodism is produced, or irritation of the stomach occurs, provided the symptoms do not yield earlier. It may be necessary to push it to 350 or 400 grains a day before the symptoms yield.

RHEUMATIC HEADACHE.

The headache of rheumatism is characterized usually by tenderness of the scalp, which is increased on pressure or motion. Use the mild faradic current on the scalp, and internally the following:

R. Potass. iodide,
Amm. muriat. .....aa..dr.iss.
Infus. humuli...............oz.vi

M. Sig. oz.ss. four times a day in a wineglass of water.

In some cases of rheumatic headache, which have not yielded to the above treatment, I have found bromide of ammonia in twenty-grain doses every two hours effectual.

URÆMIC HEADACHE.

There is another form of headache which is of great importance as a symptom of serious disease. The pain in the head may be the first evidence you will obtain that there exists renal disease, and that you really have to deal with uræmic headache. The judicious plan of treatment in such cases has for its object the removal of the abnormal amount of urine from the system. To accomplish this, you may call into action one or all of the three great emunctories of the body, the kidneys, the intestines and the skin. Make the kidneys act if you can; apply dry cups over the region of them, and give internally the following:

R. Potass. acetat............dr.vi.
Infus. digitalis............oz.vi.
M. Sig. oz.ss. q. 3 h.

The infusion should be made from fresh English leaves. Give this until the kidneys act freely, if you can make them do it within twenty-four hours. You cannot always rely on this, however. If the kidneys do not act freely and the headache is not relieved within twenty-four hours, give a saline cathartic. A treatment almost domestic, and often very effectual, is to put an ounce of cream-tartar in a quart of water, and have the patient drink this in eight or ten hours.

### ALCOHOLIC HEADACHE.

The headache of acute alcoholism, or inebriety, follows a debauch. The first indication is to remove the alcohol from the intestinal canal. For this give of rhubarb and magnesia calcined each half a drachm, then give as follows:

R. Spts. amm. aromat.........dr.ij.
Tinct. camph..............dr.iss.
Tinct. hyosciami.........dr.iiss.
Spts. lav. comp. q.s.ad...oz.ij.

M. Sig. dr.j. q. 1 h. until the headache is relieved, and then give capsicum gr.ij. and quinine gr.iij. before each meal for several days. If there be sleeplessness give:

R. Sodii bromid.............oz.ss.
Chloral hydrat........dr.iiss.
Syr. aur. cort ...........oz.ss.
Aquæ .........................oz.iiiss.
M. Sig. oz.ss. at night, repeat in two hours if necessary to produce sleep.

### DYSPEPTIC HEADACHE.

Dyspepsia is a frequent cause of headache.

If there is indigestible food in the stomach, and it has been there for some time, give an emetic, as mustard and warm water, or sulphate zinc gr. xv., and remove it. If there is evidence of indigestible food in the alimentary canal, beyond the stomach, give gr.xx. of rhubarb and magnesia each, and remove it from the bowels. If the headache be frontal, and the pain is located immediately over the eyes, give dilute nitro-muriatic acid in ten-drop doses, well diluted, after meals. If the pain is located about the roots of the hair, give an alkali before meals, as gr.xx. bicarbonate of soda or magnesia. The dyspeptic headache oftentimes is not confined to these regions, but spreads over the entire head. In such cases I combine an acid with an alkali, and add to these nux vomica, as in the following prescription:

R. Sod. bicarb.............dr.iiss.
Ac. nitro-mur. dil......dr.ij.
Tinct. nuc. vom........dr.iss.
Syr. aurant. cort........dr.vi.
Aquæ, q. s. ad.........oz.vi.

M. Sig. oz.ss. after meals in a wine-glass of water.

If there be gastric pain, a mild counter-irritant, as a mustard plaster to the epigastrium, will often relieve the pain in the head as well as the pain in the stomach. If flatulence be a troublesome symptom, give the following:

R. Bismuth subcarb........ dr.iss.
Tinct. nuc. vom ........ dr.iss.
Tinct. card. co.,
Spts. lav. comp. aa q.s.ad.oz.iv.

M. Sig. dr.ij. before meals in a wineglass of water.

If there be constipation, the following pill may be given, one in the morning:

R. Aloes pulv..................dr.ss.
Ext. nuc. vom..............gr.v.
Ext. belladonna............gr.iv.
M. Div. in pil. No. xv.

In some forms of headache associated with stomach indigestion, I have found small doses, often repeated of tinct. nux vomica effectual. I give a single drop every fifteen minutes, and continue this two or three hours if necessary. In other cases, where the headache comes on soon after a meal and seems to depend on stomach digestion large doses of pepsin are effectual. Give a half drachm saccharated pepsin in a wineglass of sherry wine, t. i. d., and let it be taken during meals.

HEADACHE FROM CONGESTION.

Cerebral congestion as a cause of headache may be divided into two varieties, active and passive. These claim almost directly opposite plans of treatment. In the active variety the patient should be kept in a darkened room, perfectly quiet, cold and evaporating lotions applied to the head. A saline cathartic may be giuen, and the following prescription :

R. Sodii bromid.............dr.iiss.
Fl. ext. ergot...........dr.iiss.
Syr. zinzib.................oz.ss.
Aq. aurant. Flor. q.s.ad. oz.iv.
M. Sig. oz.ss. q. 2 h.

If the skin be hot and dry, and the pulse full and rapid, give Fleming's Tinct. Aconit. Rad. gtt. ii. q. 2 h. until the heart's action is sensibly diminished. Sometimes a hot mustard foot-bath will give relief.

The passive congestion variety demands a different mode of treatment. In many cases this variety is found associated with cardiac disease, and most frequently where there is cardiac dilatation. Hypertrophy gives rise to the active variety. Improve the condition of the blood by the use of iron, quinine, bitter tonics, alcoholic stimulants, good food, and stimulate the heart's action by the use of the following:

R. Tinct. digitalis..............dr.iii.
Spts. amm. aromat......dr,vi.
Spts. lavand co.,
Syr. simp. aa q. s. ad.....oz.iii.
M. Sig. dr.i. q. 4 h.

ANÆMIC HEADACHE.

Cerebral anæmia produces a headache, which is often mistaken for the passive cerebral congestive form. It is often associated with general anæmia, nervous exhaustion, and may occur in heart disease in consequence of enfeebled heart-power, such as is met with in enlargement with dilatation, fatty degeneration, and myocarditis. Improve the general condition of the patient, and stimulate heart's action as recommended in the passive cerebral congestive variety. Nitrite of amyl will relieve the immediate headache. Let the patient inhale three to five drops of it on a piece of cotton, placed within one nostril while the other is held closed. When associated with nervous exhaustion, I employ the following:

R. Strych. sulph.................gr.ss.
Tinct. fe. chlor...............dr.ij.
Glycerinæ..... .............oz.ss.
Infus. gentian q. s. ad....oz.vj.
M. Sig. oz.ss. after meals, in a wineglass of water.

A word as to alcoholic stimulants. These are beneficial in headache dependent on cerebral anæmia. Champagne is a specially favorite form, and is much relished by those who suffer from nervous exhaustion. You should use caution in recommending it to such patients, as it may lead to serious results. Give it always as a remedy, and not as beverage. A safe plan is to recommend brandy, a tablespoonful after each meal, and limit the champagne to one glass, and let it be taken with the dinner.—*Western Lancet.*

